# Complete Genome Sequences of Streptococcus parauberis Serotype Ia, Ib/Ic, and II Isolates from Diseased Olive Flounder in South Korea

**DOI:** 10.1128/mra.01058-22

**Published:** 2023-01-19

**Authors:** Yoonhang Lee, Ahran Kim, Nameun Kim, HyeongJin Roh, Diem Tho Ho, Do-Hyung Kim

**Affiliations:** a Department of Aquatic Life Medicine, Pukyong National University, Busan, Republic of Korea; University of Rochester School of Medicine and Dentistry

## Abstract

We report the complete genome sequences of three isolates of Streptococcus parauberis, representing serotypes Ia, Ib/Ic, and II, which were isolated from diseased olive flounder (Paralichthys olivaceus) in South Korea.

## ANNOUNCEMENT

Streptococcus parauberis is one of the most important bacterial pathogens in olive flounder, starry flounder, and turbot (1–3). Of the seven currently proposed serotypes (3), piscine isolates derived from South Korea and Japan belonged to serotypes Ia, Ib/Ic, and II (4, 5). Here, we report the complete genome sequences of three S. parauberis isolates, i.e., HFTC0064 (serotype Ia), KSP20 (serotype Ib/Ic), and KSP10 (serotype II), which were derived from diseased olive flounder in 2014, 2005, and 2004, respectively, in South Korea.

Bacteria were isolated from the spleen of diseased olive flounder that had been sampled from commercial fish farms located off the coasts of Pohang (HFTC0064) and Jeju Island (KSP10 and KSP20), South Korea. Bacterial identification and serotyping were conducted by PCR analysis as described previously (6, 7). Prior to sequencing, bacteria were stored at −80°C in brain heart infusion (BHI) broth (Oxoid, UK) supplemented with 15% glycerol. A single colony of each S. parauberis strain grown on BHI agar plates was inoculated into BHI broth and incubated at 28°C for 18 h. Genomic DNA was extracted using the Wizard genomic DNA purification kit (Promega, USA) and quantified using a Qubit 3.0 fluorometer (Invitrogen, USA) and a Qubit double-stranded DNA (dsDNA) high-sensitivity (HS) kit (Invitrogen) according to the manufacturers’ instructions. Libraries were prepared by using a SMRTbell template preparation kit v1.0 (Pacific Biosciences [PacBio], USA) and following the standard protocol for 20-kb template preparation using the BluePippin size selection system. Whole-genome sequencing was performed with a RS II sequencer (PacBio). Read quality control (minimum subread length, 500 bp; minimum polymerase read quality, 0.80), adaptor trimming, and *de novo* assembly were performed using the Hierarchical Genome Assembly Process v3 (HGAP3) pipeline in single-molecule real-time (SMRT) Analysis v2.3.0. The circularity of contigs was confirmed using Unicycler v0.4.9 with the default settings (8). Genes were predicted and annotated based on the National Center for Biotechnology Information (NCBI) Prokaryotic Genome Annotation Pipeline (PGAP) v6.2 (9). Average nucleotide identity (ANI) analysis was conducted in EDGAR v3.0 (10). For comparison, 23 previously sequenced S. parauberis genome sequences were obtained from the NCBI database (https://www.ncbi.nlm.nih.gov/data-hub/genome/?taxon=1348), and serotyping was conducted by *in silico* PCR using the NCBI Primer-BLAST online tool (11).

The sequencing and annotation results are summarized in Table 1. Circular chromosomes with sizes of 2,081,310 and 2,169,433 bp were assembled for KSP10 and KSP20, respectively, while HFTC0064 harbored a 12,786-bp circular plasmid in addition to a circular chromosome. Among the 23 S. parauberis genome sequences that were publicly available, only serotype Ia strains, including strains SPOF3K, KRS-02083, and KCTC11980BP, harbored the identical plasmid sequence found in HFTC0064. The plasmid contained 12 coding sequences (CDSs), including a tetracycline resistance gene (*tetS*) and a virulence-related gene (*inlJ*), as described previously (12). HFTC0064, KSP20, and KSP10 shared >99.1% ANI with one another and >98.7% ANI with the genomic sequences of the 23 S. parauberis strains used for comparison. The genomic information for S. parauberis strains belonging to three different serotypes will contribute to greater understanding of the genetic diversity of this microbe.

**TABLE 1:** Summary statistics for whole-genome sequences

Strain	No. of PacBio reads	Read *N*_50_ (bp)	Genome coverage (×)	No. of contigs^*a*^	Total length (bp)	GC content (%)	No. of CDSs	No. of rRNAs	No. of tRNAs
HFTC0064	91,497	14,316	586.2	2	2,159,181	35.6	2,134	18	69
Chromosome				1	2,146,395	35.7	2,121	18	69
Plasmid				1	12,786	34.3	13	0	0
KSP20	91,791	13,796	583.6	1	2,169,433	35.6	2,210	18	70
KSP10	122,034	14,027	615.1	1	2,081,310	35.6	2,014	18	68

a Chromosome and plasmid information is shown separately when there is more than one closed contig.

### Data availability.

The complete genome sequences in this project have been deposited in GenBank under accession numbers CP104047, CP104048, CP104190, and CP104191, BioProject accession number PRJNA876642, and BioSample accession numbers SAMN30668769, SAMN30668770, and SAMN30668771.
